# Anemia Across Lifespans in Rural South India: A Comprehensive Study of Age and Gender Dynamics From the Field Practice Area of Centre for Rural Health (CRHA), Nutakki, Andhra Pradesh

**DOI:** 10.7759/cureus.77963

**Published:** 2025-01-25

**Authors:** Gowtham Sai Ambati, Abhinand ES, Akheel Ahammed PT, Rajeev Aravindakshan, Siva Santosh Kumar Pentapati, Arti Gupta

**Affiliations:** 1 Department of Community and Family Medicine, All India Institute of Medical Sciences, Mangalagiri, Mangalagiri, IND

**Keywords:** anemia prevalence, hemoglobin levels, nutritional interventions, public health burden, rural health

## Abstract

Background: Anemia is a significant public health issue in rural India, with a very big impact on health and productivity. This study investigates the prevalence and severity of anemia stratified by age and gender in a village in Southern India, using different guidelines.

Methods: Retrospective data (n=3523) from a 2023 anemia survey done in the field area at Chirravuru under the Centre for Rural Health AIIMS (CRHA), Primary Health Centre (PHC), Nutakki (a peripheral unit of All India Institute of Medical Sciences {AIIMS}, Mangalagiri) covering 90% of the estimated population of the village was taken for the study. The hemoglobin levels were estimated from capillary blood, from where anemia severity was determined. Analysis was carried out to evaluate variations by age and gender to obtain statistical inferences.

Results: Prevalence of anemia in the whole population was 79.3% (n=2792) with 32.9% (n=1159) of it being mild, 41.07% (n=1447) moderate, and 5.3% (n=186) severely anemic. Men had higher anemia of milder forms (43.3%) (n=746) than women (23.0%) (n=413), whereas women presented with a more severe form (7.8%) (n=140) compared to men (2.7%) (n=46). Moderate anemia tended to be similar in prevalence across both genders and was commoner among children under five (60%) (n=12) and adolescents (46.6%) (n=41) in comparison with adults (40.7%) (n=1350). Severe anemia was more frequent in adults (5.4%) (n=184).

Conclusions: Our findings revealed a relatively higher burden of anemia among women and children in Chirravuru. Similar areas of our country need targeted interventions to improve the hemoglobin status of adults and children through nutritional supplementation and health education.

## Introduction

Anaemia is a significant global health problem that has a big impact on both personal health and economic productivity, especially in poorer nations [1]. Over 1.62 billion people worldwide suffer from anemia, with low- and middle-income nations bearing the brunt of this burden, according to the World Health Organization (WHO) [2]. A study in 2024, reported latest Indian National Family Health Surveys data (2019-21) show a higher frequency of anemia than in 2015-16, which is alarming [3]. Anemia is very common in India, primarily in rural areas, and it is even more noticeable among vulnerable groups including women, children, and the elderly [4].

The National Family Health Survey-5 (NFHS-5) is a critical tool in understanding public health challenges in India's rural villages, focusing on anemia prevalence. Conducted by the Ministry of Health and Family Welfare, Government of India, NFHS-5 provides granular data that illuminates the socio-economic and regional disparities in anemia rates among rural populations. This makes it a vital resource for developing targeted health interventions [3]. Differences in anemia categorization between NFHS-5 [5] and WHO 2024 [6] guidelines can significantly impact reported anemia prevalence in rural areas, influencing policymakers. If WHO's criteria reveal higher anemia rates compared to NFHS-5, prompting urgent policy action. This can lead to increased resource allocation and targeted interventions to address anemia in vulnerable rural populations. Conversely, if prevalence appears lower, it might suggest successful existing measures. Aligning with WHO standards ensures international credibility and potential funding, while accurate categorization aids in designing effective programs. Understanding these differences is crucial for crafting informed and impactful public health policies.

Even though this problem has received attention, relatively little is known about how prevalent and serious it is in rural areas, where access to medical treatment and nutritional resources can be limited [7]. Past studies have mostly concentrated on urban populations [8] or general national statistics, with scanty information available on rural areas like Chirravurru, a village located in South India. Despite the anemia rates being known to be high in India, little research has looked at the problem in such a particular rural environment or investigated how it varies by age and gender [9,10]. The current study sought to fill this gap by providing a comprehensive analysis of the prevalence and severity of anemia according to the new guidelines given by WHO (2024) [6] and that adopted by NFHS-5 in Chirravuru, a village in Andhra Pradesh, South India stratified by age and gender. This study is especially beneficial since it tackled a significant public health concern in an entire village and offers crucial details that could guide focused initiatives that mitigate the impact of anemia.

## Materials and methods

This study is a retrospective data analysis conducted using data collected during an anemia survey done from January to March 2023 as a routine by the Centre for Rural Health AIIMS (CRHA), Primary Health Centre (PHC), Nutakki (a peripheral unit of All India Institute of Medical Sciences {AIIMS}, Mangalagiri) in its field practice area in Chirravuru. Chirravurru village is in Tadepalli mandal (sub-district) of Guntur district in Andhra Pradesh, India. It is situated 10 kilometers away from the sub-district headquarters Tadepalli (Tahsildar office) and 30 kilometers away from the district headquarters, Guntur.

The total geographical area of the village is 558 hectares. The population of Chirravuru village was 3702, as per Census 2011 [11]. Taking a projected increase of 7.05% and net migration of -0.03% from the population projections by the Census of India, the estimated population for 2024 was 3962 [12]. In our study, we have covered a population of 3523 (approximately 90%) of the estimated population. Every person in the village as per Accredited Social Health Activist (ASHA) books/Health and Demographic Surveillance System (HDSS) of CRHA Nutakki was contacted to participate in the anemia screening camp. Where individuals were not able to participate in the camp, house-to-house visits were made to screen for anemia. Days of the camp were planned prior, and the same was informed before all villagers. As an outreach activity under CRHA, the Nutakki anemia camp was organized with a medical officer (MO), interns, ASHAs, a female nursing orderly (FNO), a male nursing orderly (MNO), and 20 gram (village) volunteers. For the villagers who attended the camp, hemoglobin (Hb) was checked by Mission Hemoglobinometer (ACON Biotech (Hangzhou) Co. Ltd., China), which works on the principle of reflectance photometry using capillary blood. Sensitivity and specificity in detecting anemia from venous samples were 98.8% and 73.4% respectively. The positive predictive value (PPV) was 58.5%, while the negative predictive value (NPV) was 99.4% [13]. The Mission Hemoglobinometer was calibrated periodically including regular quality control checks using standardized calibration solutions as specified by the manufacturer.

The results were given out on the spot to all the villagers and the data was entered into the registers maintained under CRHA Nutakki, with those who are anemic prescribed according to Anemia Mukt Bharat guidelines [14] provided by the Ministry of Health and Family Welfare, Government of India. Those who are diagnosed with anemia are provided health information on lifestyle changes and major emphasis is kept on nutritional aspects of anemia, whereas those who are severely anemic are referred to CRHA Nutakki or the district hospital, Tenali, Guntur district, for further care and management. For those who were missed during the camp, a mop-up house-to-house survey was conducted by interns.

The data was collected from registers and no personal identifiers were retrieved from the database. Inclusion criteria encompassed all villagers registered in the ASHA books or the HDSS of CRHA Nutakki. Exclusion criteria included individuals who did not provide capillary blood samples, and villagers absent during both the camp and follow-up surveys. 

Anemia severity is stratified by age and gender using two different guidelines, NFHS-5 cutoffs (Table 1) and WHO 2024 guidelines (Table 2) [5,6].

**Table 1:** National Family Health Survey (NFHS-5) classification of anemia.

Age groups	No anemia	Mild anemia	Moderate anemia	Severe Anemia
Children 6-59 months of age	≥11 g/dL	10-10.9 g/dL	7-9.9 g/dL	<7 g/dL
Children 5-11 years of age	≥11.5 g/dL	11-11.4 g/dL	8-10.9 g/dL	<8 g/dL
Children 12-14 years of age	≥12 g/dL	11-11.9 g/dL	8-10.9 g/dL	<8 g/dL
Non-pregnant women (15 years of age and above)	≥12 g/dL	11-11.9 g/dL	8-10.9 g/dL	<8 g/dL
Pregnant women	≥11 g/dL	10-10.9 g/dL	7-9.9 g/dL	<7 g/dL
Men	≥13 g/dL	11-12.9 g/dL	8-10.9 g/dL	<8 g/dL

**Table 2:** Hemoglobin cutoffs to define anaemia severity in individuals according to World Health Organization (WHO) 2024 guidelines.

Classification of anemia based on hemoglobin	Normal	Mild	Moderate	Severe
Children, 6–23 months	≥105 g/L	95–104 g/L	70–94 g/L	<70 g/L
Children, 24–59 months	≥110 g/L	100–109 g/L	70–99 g/L	<70 g/L
Children, 5–11 years	≥115 g/L	110–114 g/L	80–109 g/L	<80 g/L
Children, 12–14 years, nonpregnant girls	≥120 g/L	110–119 g/L	80–109 g/L	<80 g/L
Children, 12–14 years, boys	≥120 g/L	110–119 g/L	80–109 g/L	<80 g/L
Adults, 15–65 years, nonpregnant women	≥120 g/L	110–119 g/L	80–109 g/L	<80 g/L
Adults, 15–65, years men	≥130 g/L	≥130 g/L	80–109 g/L	<80 g/L

All the data was entered in Microsoft Excel Office 2019 (Microsoft Corp., Redmond, WA). Data was analyzed using the IBM SPSS Statistics for Windows, Version 28 (IBM Corp., Armonk, NY). Categorical variables were summarized as frequencies and proportions. The chi-square test was used to determine the significance between the presence of anemia with age and gender, with a p-value of less than 0.05 considered statistically significant. Ethical approval and waiver of consent for the study were obtained from the Institutional Ethics Committee, All India Institute of Medical Sciences (AIIMS), Mangalagiri (Approval No. AIIMS/MG/IEC/2024-25/44, dated September 19, 2024).

## Results

Demographics and hemoglobin levels

The study covered 3523 inhabitants of the village, Chirravuru, comprising 1798 women (51.0%) and the rest men (1725; 49.0%). The mean age of the study participants was 35.7 (+11.96) years. Hemoglobin levels averaged at 10.86 (+1.71) g/dL. Women had a mean Hb level of 10.49 (+1.71) g/dL, lower than the men (11.24 +1.74 g/dL), with median levels of 10.8 g/dL for women and 11.2 g/dL for men. The values ranged from a low of 4.0 to 19.9 g/dL in the whole population.

Prevalence of anemia by severity

Using WHO (2024) anemia guidelines, the overall prevalence of anemia in the study population was 79.3%, with only 20.7% categorized as having normal Hb levels and found similar results even with NFHS-5 guidelines which were 20.72% of the whole population as not anemic.

Gender-wise categorization of anemia

**Figure 1:**
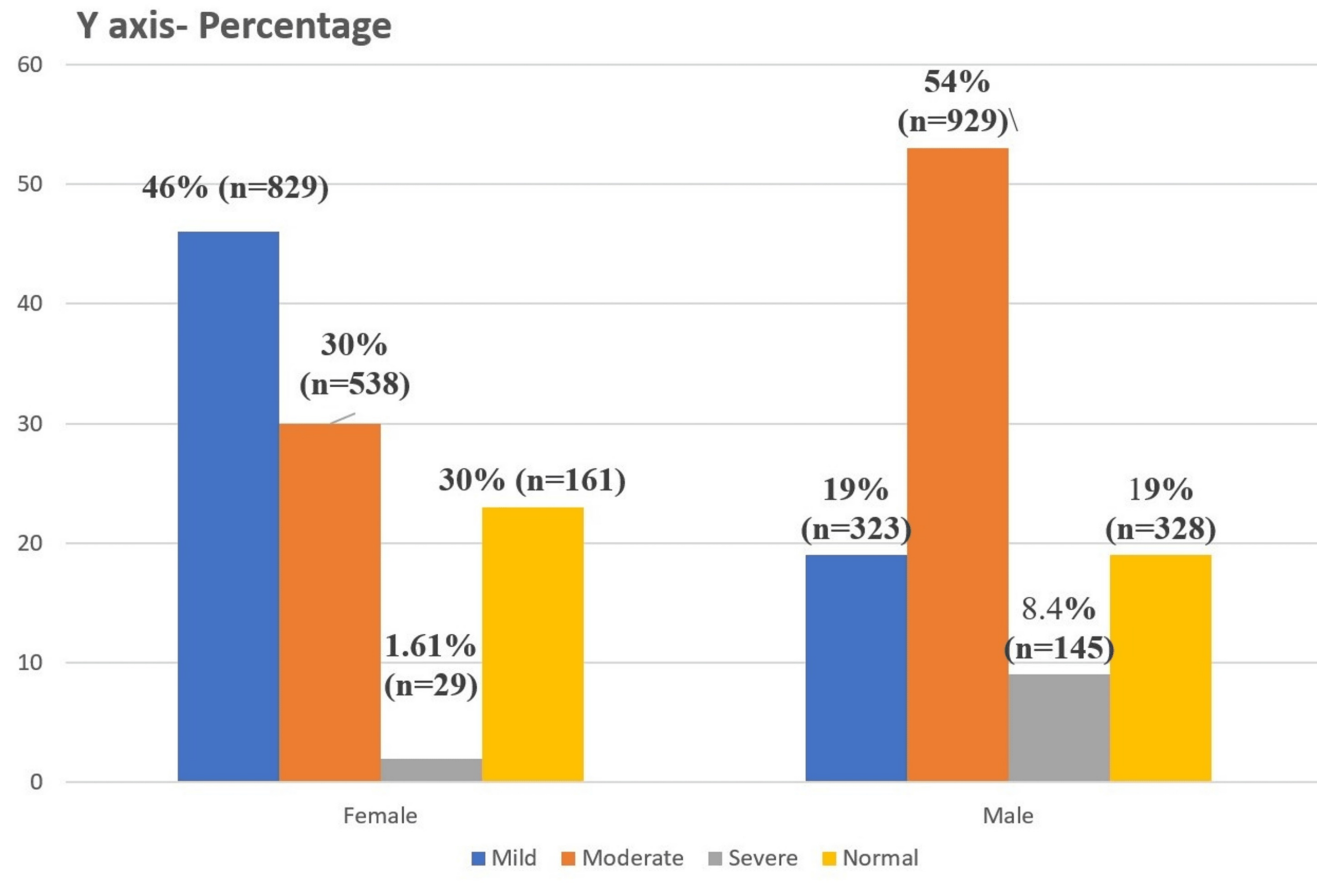
Gender-wise distribution of anemia according to NFHS-5 guidelines (n=3523). The severity of anemia is based on classification in Table 1. NFHS: National Family Health Survey.

Under NFHS-5 guidelines (Figure 1), women showed higher mild anemia (46.1%) compared to men (18.7%), while men had more moderate anemia (53.9%) than women (29.9%). Severe anemia was higher in men (8.4%) than in women (1.6%). Women showed a higher percentage of mild cases and lower severe cases compared to men in NFHS-5 with a significant difference in the severity of anemia between the two genders with p-value (p<0.01).

**Figure 2:**
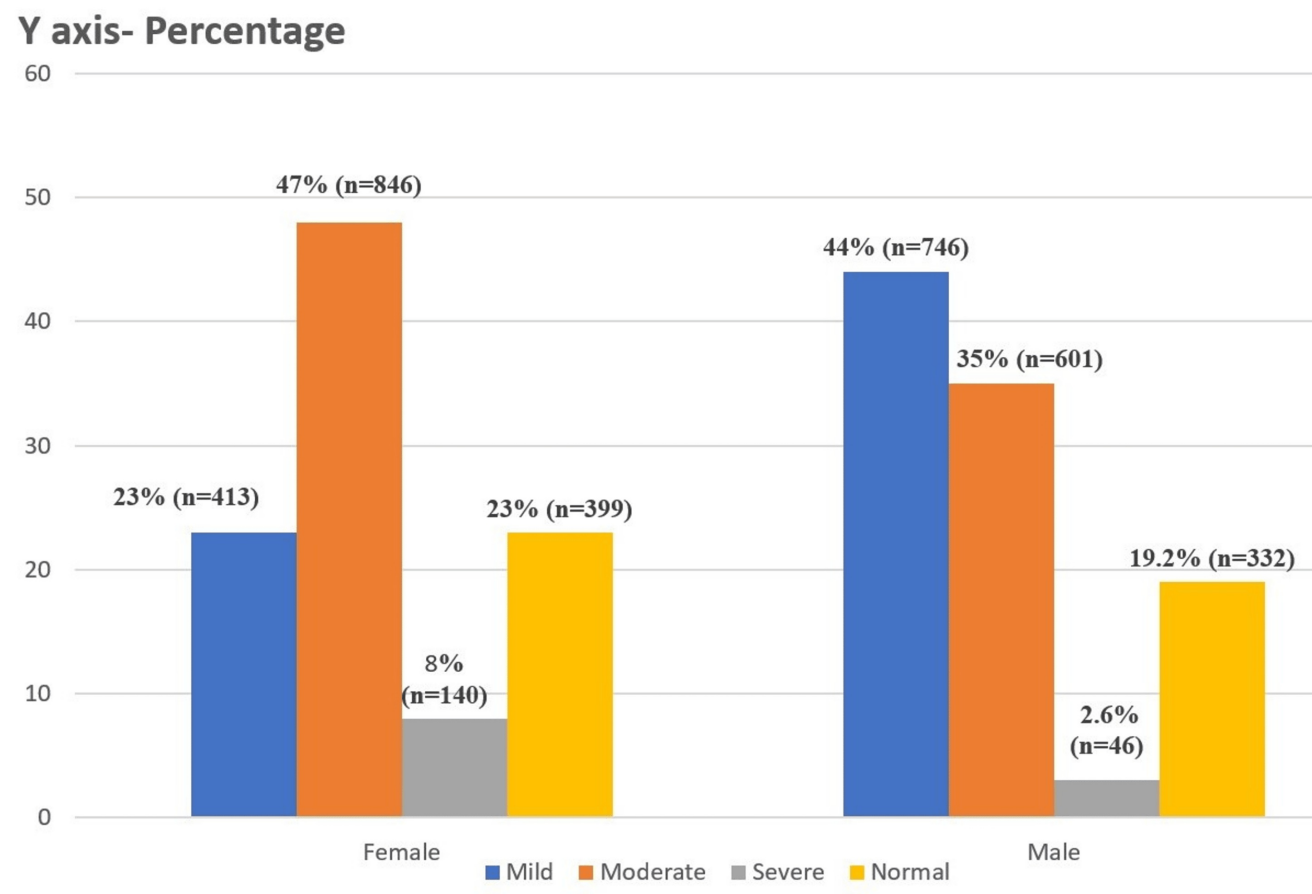
Gender-wise distribution of anemia according to WHO 2024 guidelines (n=3523). The severity of anemia is based on the classification in Table 2. WHO: World Health Organization.

Using WHO (2024) guidelines (Figure 2), moderate anemia was most prevalent in both genders (47.1% in women, 34.8% in men). Men had higher mild anemia (43.3%) than women (23.0%), but severe anemia was more pronounced in women (7.8%) than in men (2.7%). WHO anemia categories indicate more moderate anemia cases in women with a statistical significance with a p-value of <0.01.

Age-wise distribution of anemia

There is a significant difference in the distribution of anemia among different age categories as per NFHS-5 criteria (p-value: 0.034). Among all the anemia categories, the majority of the participants belonged to the middle-aged category. Details of the distribution are presented in Table 3.

**Table 3:** Distribution of anemia categories and age categories as per NFHS-5. NFHS: National Family Health Survey.

Anemia categories	Age categories						
	Adolescent	Geriatric	Middle-aged	Primary children	Under five	Total	p-value
Mild	30 (2.6)	44 (3.82)	1060 (92.01)	14 (1.22)	4 (0.35)	1152	0.034
Moderate	41 (2.79)	53 (3.61)	1350 (92.02)	11 (0.75)	12 (0.82)	1467	
Normal	17 (2.33)	19 (2.6)	675 (92.47)	15 (2.05)	4 (0.55)	730	
Severe	0	3 (1.72)	171 (98.28)	0	0	174	

There is no significant difference in the distribution of anemia among different age categories as per WHO criteria (p-value: 0.051). Among all the anemia categories, the majority of the participants belonged to the adult category. Details of the distribution are given in Table 4.

**Table 4:** Distribution of anemia categories and age categories as per WHO. WHO: World Health Organization.

Anemia categories	Age categories n (%)							
	Adult	Children (0-24 months)	Children (24 months-5 years)	Children (6-11 years)	Female children (12-14 years)	Male children (12-14 years)	Total	p-value
Mild	1138 (98.19)	1 (0.09)	3 (0.26)	6 (0.52)	4 (0.35)	7 (0.6)	1159	0.051
Moderate	1389 (95.99)	4 (0.28)	8 (0.55)	33 (2.28)	4 (0.28)	9 (0.62)	1447	
Normal	704 (96.31)	0	4 (0.55)	18 (2.46)	3 (0.41)	2 (0.27)	731	
Severe	184 (98.92)	0	0	2 (1.08)	0	0	186	

A slight difference was observed between the categories of anemia using two guidelines. WHO (2024) classification showed a higher prevalence of severe anemia (5.3%) compared to NFHS-5 (4.9%). Moderate anemia however showed almost similar prevalences using both the guidelines, i.e., WHO (41.1%) than NFHS-5 (41.6%) (Table 5).

**Table 5:** Comparison of anemia severity by WHO and NFHS-5 guidelines (n=3523). WHO: World Health Organization, NFHS: National Family Health Survey.

Anemia severity	WHO	NFHS-5
	n (%)	n (%)
Mild	1159 (32.9)	1152 (32.7)
Moderate	1447 (41.1)	1467 (41.6)
Severe	731 (5.3)	730 (4.9)
Normal	186 (20.7)	174 (20.7)

## Discussion

This study presented a routine evaluation of the hemoglobin status of a village in rural Andhra Pradesh, which revealed the high prevalence and severity of anemia among the residents. The study revealed a concerning anemia prevalence of 79.3% among the population, using WHO (2024) guidelines, with only 20.7% having normal hemoglobin levels. The NFHS-5 guidelines corroborate these findings, showing 20.72% as non-anemic. With Chirravuru village's population estimated at 3962 in 2024, based on Census 2011 data and subsequent projections, our study covered approximately 90% of this population, equating to 3523 individuals. The anemia, affecting every household with an average household size of four [15], underscores the urgent need for innovative intervention strategies.

The study found that 79.3% of the population was anemic, aligning with previous findings in other rural Indian populations where anemia prevalence ranges between 50% and 80% [16,17]. Secondly, women had higher rates of severe anemia (7.8%) than men (2.7%), which is in line with previous research studies done in rural India [18] emphasizing these gender variations can be due to menstrual blood loss, pregnancies, and limited nutritional resources for women in rural India [19,20]. This high prevalence highlights the need for improved nutritional resources and the availability of accessible health care through various health schemes for rural women in India. The analysis highlights significant insights into anemia prevalence across age groups, notably aligning with NFHS-5 results, which provide detailed age categorization, including geriatric groups absent in WHO 2024 guidelines. Moderate anemia is prevalent across all ages, with under-five children (60%) and adolescents (46.1%) most affected. This underscores inadequate awareness of exclusive breastfeeding and complementary feeding in rural India. Anemia among women and children in rural India remains a pressing public health issue, with various cultural and behavioral factors contributing to its prevalence. Dietary patterns often lack diversity, with staple diets predominantly comprising cereals and lacking essential iron-rich foods, exacerbating anemia risk [21]. Cultural practices, such as early marriage and subsequent pregnancies, further increase anemia incidence among women due to insufficient recovery periods between childbirths [22]. Additionally, limited autonomy in health-related decision-making can prevent women from accessing nutritional supplements or healthcare services, perpetuating poor health outcomes [23]. Addressing these gaps requires targeted educational initiatives to promote proper nutritional practices. Empowering caregivers and communities through awareness programs can play a crucial role in mitigating anemia and improving health outcomes across diverse age demographics in rural settings.

The geriatric population's anemia prevalence is alarming, with only 15.9% having normal hemoglobin levels, highlighting critical issues like inadequate healthcare access [24], nutritional shortages, and the absence of tailored policies. Comprehensive geriatric assessments and full hematological evaluations are essential, as the predominant anemia causes include anemia of chronic disease (41.67%), iron deficiency anemia (35%), and others like myelodysplastic syndrome and megaloblastic anemia [25]. These findings underline the urgent need for targeted interventions and public health strategies addressing anemia at both age spectrum extremes. Effective anemia management can substantially reduce mortality and morbidity, enhancing the quality of life for vulnerable populations.

The WHO (2024) guidelines report slightly higher severe anemia rates (5.3%) compared to NFHS-5 (4.9%), likely due to lower thresholds for classification. However, these guidelines fail to classify geriatric anemia prevalence specifically, indicating a gap in age-specific assessments. Additionally, notable gender differences emerge between WHO and NFHS-5 classifications. Aligning our classification with previous research underscores the need for unified diagnostic criteria to standardize anemia assessment globally. Such standardization would enhance the accuracy and comparability of anemia data, facilitating better-targeted interventions and policies to effectively address this public health challenge.

The widespread anemia among women, children, and the elderly underscores an urgent need for public health action. Initiatives like India's "Anemia Mukt Bharat" campaign [14], which enhances iron and folic acid supplementation, deworming, and nutritional education, offer promising frameworks to curb anemia rates. However, tailored programs focusing on the elderly are crucial for addressing grassroots-level anemia. Community-based interventions should be integrated into national health programs, especially in rural areas, to overcome challenges such as inadequate healthcare infrastructure and cultural beliefs impeding nutritional progress. These targeted strategies can enhance resource accessibility and foster significant improvements in anemia management.

Current public health initiatives must be re-evaluated to incorporate more comprehensive approaches that address underlying socioeconomic and nutritional determinants of anemia. Community engagement, particularly in rural settings, can foster culturally sensitive educational programs to improve dietary practices and healthcare access. Moreover, integrating technology and leveraging local resources to ensure consistent iron and folic acid supplementation could enhance intervention effectiveness [26]. Research should also focus on understanding the barriers to anemia prevention, such as food insecurity and healthcare accessibility, to tailor strategies that are both sustainable and impactful. By adopting a holistic approach, public health policies can more effectively combat anemia and improve overall health outcomes in Chirravuru village and similar communities. Effective communication strategies, including community-based educational programs, can play a crucial role in promoting dietary changes [27]. Emphasizing the importance of iron-rich foods, such as leafy greens, legumes, and fortified cereals, can help improve nutritional practices. Additionally, leveraging local media and healthcare workers to disseminate information about affordable and accessible iron-rich food options can facilitate behavioral change. Incorporating traditional knowledge and practices that align with cultural dietary preferences may also enhance the acceptance and sustainability of these interventions. By fostering community involvement and using culturally resonant communication, public health initiatives can better address anemia's root causes and improve health outcomes in Chirravuru and similar rural settings. Addressing the high anemia prevalence in Chirravuru requires specific strategies. Initiating local food fortification programs can enhance the nutritional value of staples like flour and rice with iron and folic acid. Collaborating with Anganwadi centers for regular anemia screening and education is crucial. These centers can conduct routine hemoglobin tests and provide nutritional workshops, ensuring early detection and intervention. Community engagement is vital; using culturally resonant communication and involving local leaders can boost awareness and participation. These targeted actions aim to significantly reduce anemia rates, improve health outcomes, and enhance the community's overall well-being and productivity.

Despite achieving extensive population coverage, the study has limitations. Hemoglobin levels were determined using capillary blood samples, which may not perfectly align with venous blood measurements. Additionally, the cross-sectional design focused primarily on variables like age, gender, and hemoglobin, neglecting other critical cultural and nutritional factors influencing anemia prevalence.

Despite limitations like the cross-sectional design and capillary blood reliance, the study's robust data collection and large sample size, covering 90% of the population, underscore its validity. The use of WHO (2024) guidelines enhances its relevance, making the findings significant in guiding effective anemia intervention strategies. Longitudinal studies are essential to evaluate the long-term effects of anemia control strategies. Future research should delve into socio-economic and dietary factors contributing to anemia in rural regions, aiming to develop comprehensive intervention strategies that effectively reduce anemia-related mortality and morbidity.

## Conclusions

The study highlights an alarming anemia prevalence of 79.3% in Chirravuru village, with only 20.7% of residents having normal hemoglobin levels. These findings align with both WHO (2024) and NFHS-5 guidelines, though WHO reports slightly higher severe anemia rates due to differing thresholds. Such consistency underscores the pressing need for targeted interventions. Addressing this widespread anemia requires innovative strategies like local recipes, traditional knowledge capture, local food fortification and regular screenings. Community engagement and culturally sensitive educational programs are crucial to improving dietary practices and healthcare access. By implementing these measures, public health policies can significantly reduce anemia rates, enhancing the overall health and quality of life for the village's residents.
